# Longitudinal changes in left atrial function during chronic ibrutinib therapy: a prospective observational study

**DOI:** 10.3389/fcvm.2026.1914493

**Published:** 2026-08-26

**Authors:** Matea Kolacevic Zeljkovic, Sandra Basic-Kinda, Dubravka Čaržavec, Marijo Vodanovic, Inga Mandac Smoljanovic, Ozren Jaksic, Matea Novak, Slobodanka Ostojic-Kolonic, Dino Dujmovic, Vlatko Pejsa, Igor Aurer, Nikola Bulj

**Affiliations:** 1 Department of Internal Medicine, Sisters of Mercy University Hospital Centre, Zagreb, Croatia; 2 Division of Hematology, Department of Internal Medicine, Zagreb University Hospital Centre, Zagreb, Croatia; 3 Medical School, University of Zagreb, Zagreb, Croatia; 4 Division of Hematology, Department of Internal Medicine, Merkur University Hospital, Zagreb, Croatia; 5 Division of Haematology, Department of Internal Medicine, Dubrava University Hospital, Zagreb, Croatia; 6 Zagreb School of Economics and Management, Zagreb, Croatia; 7 Department of Cardiology, Sisters of Mercy University Hospital Centre, Zagreb, Croatia

**Keywords:** function, ibrutinib, left atrium, strain, volume

## Abstract

**Background:**

Ibrutinib is effective for B-cell malignancies but is associated with cardiovascular adverse events, including atrial fibrillation (AF) and hypertension. Longitudinal data on left atrial (LA) remodeling during treatment are limited. We characterized serial changes in LA size and function during chronic ibrutinib therapy.

**Methods:**

In this prospective observational cohort, 40 patients starting ibrutinib underwent clinical assessment, 24–72-hour Holter monitoring, and echocardiography with LA strain at baseline and 3, 6, 12, and long-term (nominal 36-month) follow-up. Primary analyses used observed paired data, random-intercept mixed models, standardized response means (SRMs), and Benjamini-Hochberg adjustment across 24 primary contrasts.

**Results:**

Median age was 65 years and 53% were female. Paired mean LAVI changes were +1.52 mL/m² at 3 months (*n* = 38), +1.68 at 6 months (*n* = 36), +2.62 at 12 months (*n* = 33), and +2.15 at long-term follow-up (*n* = 31; all q ≤ 0.020). LA reservoir strain worsened from 6 months onward (all q ≤ 0.031), while LA contractile strain, LA lateral-wall TDI, and LV GLS worsened at every follow-up (all q ≤ 0.009). Results were materially unchanged after adjustment for time-varying hypertension and in complete-case sensitivity analyses. Incident AF occurred in 5/40 patients (12.5%; 95% CI 5.5%–26.1%).

**Conclusion:**

During chronic ibrutinib therapy, longitudinal imaging showed modest LA enlargement and consistent deterioration in several LA deformation indices. These treatment-associated observations require confirmation in controlled cohorts.

**Clinical trial registration:**

ClinicalTrials.gov, identifier NCT03751410.

## Introduction

Ibrutinib, a first-in-class, irreversible (covalent) and potent Bruton tyrosine kinase inhibitor (BTKi), has revolutionized the therapeutic landscape for B-cell malignancies, significantly driving down progression-free survival risks and improving clinical outcomes (1–3). It is generally well tolerated; however, its long-term use is limited by clinically important cardiovascular toxicities (2–6). Among these, ibrutinib-related atrial fibrillation (AF) stands as the most prominent and therapy-limiting complication, exhibiting an incidence rate between 4% and 16% in real-world clinical cohorts (6–8). Beyond generating cardiovascular morbidity, including elevated risks of heart failure and systemic thromboembolism, ibrutinib-related AF routinely disrupts oncological continuity, often necessitating dose reductions or complete drug cessation (8–10). Despite its widespread clinical manifestation, the data on mechanisms underlying arrhythmias observed during ibrutinib therapy remain limited (11). Emerging basic and preclinical evidence points to off-target, non-selective kinase inhibition in cardiac tissue (11–13). Specifically, the disruption of the PI3K-Akt-mTOR signaling cascade and the inhibition of C-terminal Src kinase are highly implicated in structural remodeling, micro-inflammation, and the autophagic degradation of connexins within the atrium (14). This biomolecular remodeling is mirrored clinically by incident or worsening hypertension and progressive structural alterations (8, 14). In addition, because ibrutinib concurrently damages platelet aggregation mechanisms, managing subsequent arrhythmias with conventional anticoagulation requires a delicate, high-risk balancing act between thromboembolic prevention and severe bleeding events (15, 16).

Advanced speckle-tracking echocardiography (STE) has recently stepped to the forefront of cardio-oncology by enabling sensitive quantification of LA deformation metrics (17). Observational data suggest that chronic ibrutinib exposure may interact selectively with these subclinical mechanical properties, pointing toward a scenario where the drug acts as a metabolic precipitant on a pre-existing substrate of elevated atrial vulnerability (18). Previous studies have linked pretreatment LA enlargement and impaired LA mechanics to AF during ibrutinib therapy, but serial component-wise LA mechanics over prolonged follow-up remain incompletely characterized. We therefore aimed to describe longitudinal changes in LA size, reservoir, conduit, and contractile function during chronic ibrutinib therapy.

## Methods

### Study design and population

This was a multicenter, prospective, longitudinal, observational cohort study. Consecutive patients with B-cell lymphoproliferative disorders starting ibrutinib therapy were included across four tertiary centres: *Sisters of Mercy* University Hospital, *Zagreb* University Hospital, *Merkur* University Hospital, and *Dubrava* University Hospital. However, the specialized cardiology work-up and follow-up—including strain analysis—were performed exclusively at the *Sisters of Mercy* University Hospital.

Patients with structural heart disease, including coronary artery disease and moderate to severe valvular pathology, stroke, advanced chronic kidney disease, any clinically significant arrhythmias were excluded from the study. No patient had AF at enrolment.

This study used de-identified data, with all participants providing written informed consent for inclusion in the study. Ethical approval was officially obtained from the Ethics Committee of the *Sisters of Mercy* University Hospital, as well as from the Ethics Committee of the School of Medicine, University of Zagreb. The study was conducted in accordance with the Declaration of Helsinki and data processing complied with GDPR. Patients or the public were not involved in the study's design, conduct, reporting, or dissemination. The trial was registered at ClinicalTrials.gov with ID number: NCT03751410 - Chronic Ibrutinib Therapy Effect on Left Atrial Function (CITE-LA study).

### Follow-up

Patients' diagnostic assessment was carried out before and repeated at 3, 6 and 12 months, as well as 36-months after the introduction of ibrutinib. Each follow-up visit included demographics, clinical cardiovascular comorbidity and treatment, blood pressure, 12-lead ECG, laboratory parameters, 24–72 h Holter-ECG variables, conventional echocardiographic measures as well as speckle-tracking-derived LA strain parameters.

All included patients had a standardized transthoracic echocardiogram (Vivid 9, General Electrics, Chicago, Illinois, USA) with TDI and strain analysis. All datasets were analyzed offline using the Q-Analysis software (General Electric EchoPac workstation, version 112). One experienced cardiologist, who was blinded for the study endpoint, did the measurements as well as performed the analyses offline in a standardized manner with five consecutive measurements for each parameter in every patient.

The echocardiographic variables of interest were left atrial volume index by biplane method (LAVI), 2-dimensional left atrial (LA) volume, LA reservoir strain, LA conduit strain, LA contractile strain, LA lateral wall tissue Doppler velocity (TDI), left ventricular diastolic dis/function and left ventricular global longitudinal strain (GLS).

STE data were acquired at frame rates of 60–90 frames per second using ECG R-R gating, where the onset of the QRS complex (left ventricular end-diastole) served as the zero-strain baseline reference (19). LA strain analysis was performed using vendor-specific software [Automated Function Imaging adapted for the left atrium (AFI-LA); General Electrics, Chicago, Illinois, USA], combining apical four-chamber (A4C) and apical two-chamber (A2C) views for averaging (19). Image quality criteria required high-resolution, non-foreshortened views, appropriate frame rates (60–90 frames/s), optimized gain and compression settings, and stability across 3–5 consecutive cardiac cycles (all patients were in sinus rhythm) (19). Non-trackable segments were managed by manually adjusting the region of interest width, manually redrawing or correcting the segments, or, if necessary, discarding the acquisition and acquiring a new image (19). LVEF was calculated using the Simpson's method from the apical four chamber and the apical two chamber views (20). For 2-dimenssional LA volume quantification (in milliL - mL), modified Simpson's biplane volume equation was used (20, 21). Indexed 2D left atrial volume (LAVI, mL/m2) was calculated by dividing the estimated end-systolic LA volume with the body surface area (m2) (20, 21). TDI was performed at a frame rate of over 100 frames per second. The TDI recording at 150 ms before the QRS complex and at 800–900 ms before the QRS complex was selected for analysis of the systolic phase and diastolic phase in all segments, respectively (20–22). Because GLS, LA conduit strain, and LA contractile strain were recorded as negative strain values, their absolute values were used for longitudinal analyses, such that lower absolute values indicated worse deformation. As actual elapsed time occasionally differed from the nominal visit label, inferential analyses were indexed to the scheduled follow-up visit (3, 6, 12, and 36 months), while actual time from baseline was summarized descriptively.

Holter-ECG variables of interest were ventricular premature beats (VPBs), non-sustained ventricular tachycardia, supraventricular premature beats (SVPBs) and atrial arrhythmias lasting > 30 s. Incident AF was defined as the first documented AF diagnosis. AF incidence was assessed using a combination of symptom-driven standard 12-lead ECGs - obtained whenever patients reported symptoms suggestive of arrhythmias, supplemented by scheduled 24–72-hour Holter-ECG monitoring performed at 3, 6, 12 months, and during long-term follow-up visits. AF diagnosed between scheduled assessments was captured at the next study visit, therefore, event time was interval censored. Continuous rhythm monitoring was not used.

Incident arterial hypertension and escalation of antihypertensive therapy were also in focus. Incident arterial hypertension was defined among patients without baseline hypertension at the first follow-up visit with hypertension recorded. Antihypertensive treatment burden was quantified as the number of prescribed drug classes (ACE inhibitor, calcium-channel blocker, diuretic, and beta-blocker), and treatment escalation was defined as any increase vs. the previous visit.

### Outcomes

The primary objective was a > 10% relative decrease in LA strain deformation. Reservoir, conduit, and contractile strain were analyzed as continuous longitudinal outcomes and in a supplementary component-wise >10% decline analysis. LAVI was a central structural outcome. LA lateral-wall TDI and LV GLS were complementary remodeling measures.

Secondary end points were as follows: diastolic left ventricular function, incidence of AFib, number of ventricular premature beats (VPBs) and non-sustained ventricular tachycardia on 24–72 h Holter-ECG, and incident (newly diagnosed) or worsened hypertension.

### Data management and statistical analysis

Data were screened for internal consistency, missingness, and clinically implausible values. Primary analyses were based on observed data without interpolation or cohort-median imputation. Continuous variables are presented as mean ± SD and median (IQR), and categorical variables as *n* (%). Within-patient baseline-to-follow-up changes were assessed using two-sided Wilcoxon signed-rank tests. Paired mean changes are reported with nonparametric bootstrap 95% confidence intervals and standardized response means (mean paired change divided by the SD of paired changes). Longitudinal trajectories were additionally evaluated using linear mixed-effects models with visit as a categorical fixed effect and patient as a random intercept, a sensitivity model additionally included contemporaneous hypertension as a time-varying covariate and was not interpreted as formal mediation. For the six central remodeling measures (LAVI, LA reservoir, conduit and contractile strain, LA lateral-wall TDI, and LV GLS) across four follow-up contrasts (24 tests), Benjamini-Hochberg adjustment controlled the false-discovery rate at 5%, q < 0.05 defined statistical significance for this central inferential family. Secondary analyses are reported with nominal *p* values and interpreted as exploratory. A separate BH sensitivity analysis was performed across the 28 secondary echocardiographic contrasts. Holter-ECG count variables were summarized as median (IQR), with Friedman tests on complete cases and paired Wilcoxon tests versus baseline. Paired binary changes used exact McNemar tests, and complete longitudinal binary analyses used Cochran's Q. AF-subgroup comparisons were exploratory and used Mann–Whitney U tests with bootstrap confidence intervals for mean differences. A complete-case analysis across all five visits assessed robustness to missing data. Formal power analysis was done for LA reservoir strain of detecting differences > 10% with alpha = 0.05 and 1-beta = 0.9, which resulted in total sample size *n* = 24. We acknowledge that other LA function strain parameters are also as important as reservoir, but according to guidelines it was chosen for the power analysis (19). The trial registry records an enrollment of 40 participants. All tests were two-sided. Analyses used Python 3.11 with SciPy and statsmodels.

## Results

This longitudinal, observational cohort study included 40 patients recruited from 4 tertiary centers [median age 65 years with interquartile range (IQR) 58–72, 53% female]. The most common indication for ibrutinib treatment was chronic lymphocytic leukemia (CLL) in 38 (95%) patients, while one patient was diagnosed with Waldenström macroglobulinemia and one with Mantle cell lymphoma. Total of 33 patients (33/40) had been introduced with ibrutinib as a second, third or even fourth line of therapy. The summary of the recorded previous treatments (protocols) included: immunochemotherapy and combined protocols like - rituximab ± FCR protocol (Fludarabine, Cyclophosphamide, and Rituximab), ± CVP protocol, chlorambucil combined with rituximab, obinutuzumab-based therapies, bendamustine-based regimens and combinations (including sequences of FCR followed by bendamustine) as well as chemotherapy - chlorambucil monotherapy or combined with steroids.

The most prevalent cardiovascular comorbidity was hypertension (45%), followed by hyperlipidemia in 25%, and diabetes mellitus in 20% of patients, and no patient had AF at enrolment. Mean left ventricle ejection fraction was 60%, left atrial diameter 40 ± 4 mm and volume 53 ± 7 mL. Baseline characteristics of the study population are shown in Table 1.

**Table 1:** Baseline characteristics of the study population.

Characteristic	Total (*N* = 40)
Demographics	
Age (years)	65 (58–72)
Male sex [% (n)]	47 (19)
BMI (kg/m2)	27.5 ± 4.4
History [% (n)]	
Hypertension	45 (18)
Diabetes mellitus	20 (8)
Hyperlipidemia	25 (10)
Smoking	15 (6)
Stroke/TIA	0
Atrial fibrillation	0
Chronic kidney disease	13 (5)
COPD/Asthma	5 (2)
CHA2DS2VASc score	1.90 ± 1.55
CHA2DS2VA score	1.49 ± 1.26
HAS BLED score	0.3 ± 0.46
Hemato-oncologic disease	
Chronic lymphocytic leukemia	95 (38)
Waldenstrom macroglobulinemia	2.5 (1)
Mantle-cell lymphoma	2.5 (1)
Drugs	
Antihypertensive	38 (15)
Beta-blockers	30 (12)
Statins	13 (5)
Oral anticoagulants	5 (2)
Acetylsalicylic acid	5 (2)
Laboratory results	
Hemoglobin (g/L)	128 ± 19
White blood cell (x 109)	48 ± 42
Lymphocyte (x 109)	41 ± 41
Platelets (x 109)	157 ± 77
Urea (mmol/L)	6.5 ± 2.1
Creatinine (µmol/L)	84 ± 26
Total cholesterol (mmol/L)	5.2 ± 1.2
HDL cholesterol (mmol/L)	1.0 ± 0.45
Creatin-kinase (IU/L at 37 °C)	57 ± 40
Hs-cTnT (ng/L)	10.8 ± 4.7
NT-pro BNP (pg/L)	328 ± 294
Lactate dehydrogenase (mmol/L)	264 ± 170
C-reactive protein (mmol/L)	8.0 ± 12.9
Iron	16.8 ± 5.1

BMI, body mass index; TIA, transitory ischemic attack; COPD, chronic obstructive pulmonary disease; Hs-cTnT, high-sensitive cardiac troponin T; NT-pro BNP, N-terminal pro-B-type natriuretic peptide.

Median follow-up was 37 (IQR 23–50) months and follow-up data were available for 38, 36, 34, and 33 patients at nominal 3-, 6-, 12-, and 36-month visits, respectively. At 3 months, two participants missed the scheduled visit but subsequently returned. By 12 months, two participants had died, one could not attend because of severe disability, and three had no documented reason for missing follow-up. By long-term follow-up, one additional death had occurred. Six patients had at least one recorded reduced non-zero dose and five had at least one 0-mg/not-receiving entry.

**Figure 1:**
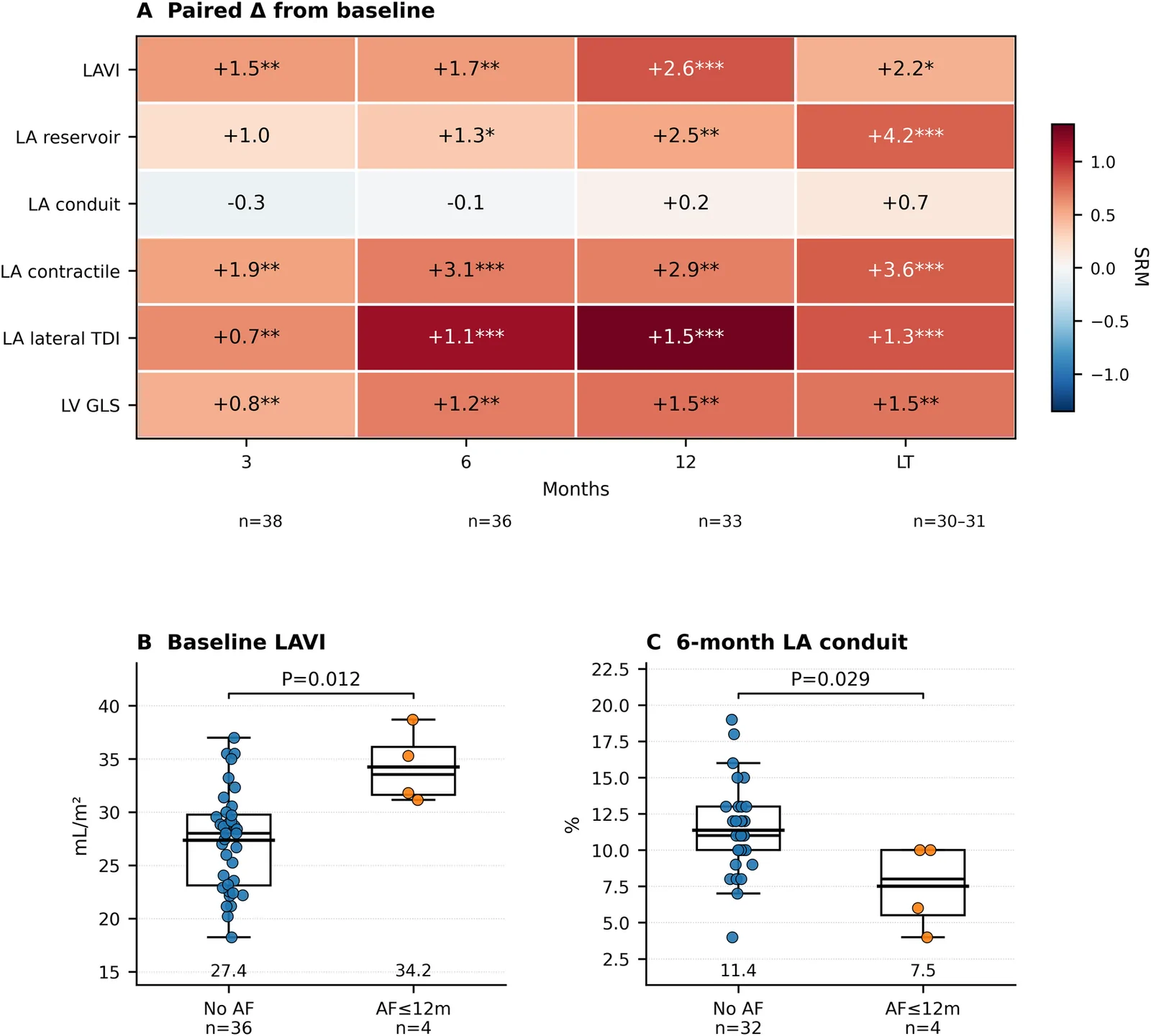
Longitudinal atrial-remodeling signal and incident-AF phenotype. Panel **(A)** shows paired mean change from baseline in the direction of worsening; color denotes standardized response mean (SRM), and cell stars denote Benjamini-Hochberg-adjusted q values from paired Wilcoxon signed-rank tests (*q < 0.05, **q < 0.01, ***q < 0.001). For LAVI, positive change indicates enlargement; for strain, TDI, and GLS, positive change indicates worsening/decrease in absolute deformation or tissue velocity. Panels **(B,C)** show patient-level distributions by incident AF by 12 months; boxes show median and IQR, points are individual patients, horizontal black lines denote means, and *P* values are from Mann–Whitney U tests. LT indicates the final available long-term visit. LAVI, left atrial volume index; TDI, tissue Doppler imaging; GLS, global longitudinal strain; IQR, interquartile range; LT, long term.

LAVI increased within paired observations at each visit: +1.52 mL/m² at 3 months (*n* = 38; pair-specific medians 28.53–29.49; q = 0.002), +1.68 at 6 months (*n* = 36; 28.00–29.62; q = 0.001), +2.62 at 12 months (*n* = 33; 27.46–31.11; q < 0.001), and +2.15 at long-term follow-up (*n* = 31; 27.46–28.50; q = 0.020). The trajectory peaked at 12 months and then plateaued. LA reservoir strain worsened from 6 months onward, while LA contractile strain, LA lateral-wall TDI, and LV GLS worsened at all follow-up visits, LA conduit strain did not change significantly (Supplementary Table S1 and Figure 1). Random-intercept mixed models confirmed the principal trajectories. At long-term follow-up, estimates versus baseline were +2.17 mL/m² for LAVI (95% CI 1.02–3.32), −3.89 points for reservoir strain (95% CI −5.30–−2.48), −3.47 points for contractile strain (95% CI −4.84–−2.11), −1.30 cm/s for LA lateral-wall TDI (95% CI −1.65–−0.94), and −1.55 points for LV GLS (95% CI −2.15–−0.95). Adjustment for time-varying hypertension produced materially similar visit estimates (Supplementary Table S2). Diastolic indices were largely stable. E/A, mitral-annular e′, and E/e′ showed no significant overall longitudinal change. Transmitral E velocity was nominally lower at the long-term (nominal 36-month) visit than at paired baseline [0.67 [0.58–0.81] vs. 0.74 [0.60–0.90] m/s; *p* = 0.041], but this did not survive secondary-family BH adjustment (q = 0.391). The prespecified probable elevated-filling-pressure/diastolic-dysfunction composite remained uncommon (5.0%, 10.5%, 8.3%, 6.1%, and 12.9% from baseline through long-term follow-up, complete-case Cochran Q *p* = 0.615) (Table 2). At 12 months, a > 10% relative decline from baseline occurred in 16/33 (48.5%; 95% CI 32.5%–64.8%) for reservoir strain, 14/33 (42.4%; 95% CI 27.2%–59.2%) for conduit strain, and 20/33 (60.6%; 95% CI 43.7%–75.3%) for contractile strain, 27/33 (81.8%; 95% CI 65.6%–91.4%) had a > 10% decline in at least one component.

**Table 2:** Echocardiographic and cardiac rhythm parameters during clinical follow-up visits.

Parameter	Baseline	3-month	*P* value	6-month	*P* value	12-month	*P* value	36-month	*P* value
LA volume (mL)	55 (48–56.25)	55 (48–56.25)	0.109	55 (45–55.75)	0.593	55 (45–58)	0.074	55 (48–60)	0.048
LAVI (mL/m2)	28.5 (23.5–31.2)	29.5 (25.3–32.5)	0.001	29.6 (24.8–33.5)	<0.001	31.1 (25.2–34.2)	<0.001	28.5 (25.2–34.9)	0.015
LA reservoir	25 (23–29)	25 (22–27)	0.103	25 (21.8–26.2)	0.024	23 (21–26)	0.007	21.6 (20–24.5)	<0.001
LA conduit	10.5 (7.8–14)	10 (9–13)	0.673	11 (9.8–12.3)	0.843	10 (9–12)	0.806	10 (7.2–11.1)	0.383
LA contractility	15 (13–17.3)	13.5 (11.3–15)	0.002	13 (10–14)	<0.001	13 (10–15)	0.001	11.8 (10–13.7)	<0.001
LA lateral wall TDI	7.7 (6.72–8.9)	7.1 (6.11–7.77)	<0.001	6.5 (6.03–7.34)	<0.001	6.1 (5.4–7.31)	<0.001	6.0 (5.46–7.29)	<0.001
LV GLS	18 (17–19)	17 (16–18)	0.006	17 (15–18)	<0.001	16 (15–18)	<0.001	16 (15–18)	<0.001
LVEF (%)	57 (55–60)	56.5 (54.25–60)	0.275	57 (54.25–59)	0.127	55 (53–58)	0.154	56 (52.5–58)	0.099
Mitral E velocity (m/s)	0.74 (0.60–0.90)	0.77 (0.69–0.86)	0.310	0.76 (0.65–0.84)	0.935	0.75 (0.65–0.80)	0.308	0.67 (0.58–0.81)	0.041
Mitral A velocity(m/s)	0.90 (0.82–0.94)	0.93 (0.78–1.0)	0.801	0.91 (0.74–1.02)	0.762	0.89 (0.82–0.99)	0.495	0.89 (0.73–0.95)	0.084
E/A	0.80 (0.74–0.99)	0.87 (0.74–0.99)	0.557	0.82 (0.72–1.01)	0.850	0.85 (0.78–0.93)	0.837	0.82 (0.74–0.89)	0.176
e′ (m/s)	8 (6–9)	8 (7–9)	0.165	8 (6.75–9)	1	7 (6–8)	0.089	7 (6–8.5)	0.394
E/e′	10.1 (7.79–11.5)	9.73 (7.74–11.0)	0.931	9.5 (8.02–11.4)	0.239	10.3 (8.70–11.4)	0.112	10.3 (7.88–12.3)	0.946
Cumulative AF incidence (n)	0	2.5 (1)	0.99	2.5 (1)	1	10 (4)	0.125	12.5 (5)	0.063
VPBs	10 (2.5–68)	20.5 (5–86)	0.647	8 (1–81)	0.852	15 (1.75–65)	0.883	20 (6–70)	0.388
SVPBs	34 (9–138)	36 (15–97)	0.525	28 (10–97)	0.915	28 (11–114)	0.688	31 (18–135)	0.178

LA, left atrial; LAVI, left atrial volume index; TDI, tissue-doppler-imaging; LV, left ventricle; GLS, global longitudinal strain; LVEF, left ventricular ejection fraction; AF, atrial fibrillation; VPB, ventricular premature beat; SVPB, supraventricular premature beat.

Incident AF occurred in 5/40 patients (12.5%; Wilson 95% CI 5.5%–26.1%). Exploratory comparisons by AF status at 12 months (AF, *n* = 4; no AF, *n* = 36) showed higher baseline LAVI in the AF group (34.23 vs. 27.35 mL/m²; mean difference +6.88, bootstrap 95% CI 3.70–10.32; Mann–Whitney *p* = 0.012). At 6 months, conduit strain was lower among patients who subsequently had AF (*n* = 4) than among evaluable AF-free patients (*n* = 32; 7.50% vs. 11.38%; mean difference −3.88 points, bootstrap 95% CI −6.66–−1.19; *p* = 0.029). These small-event analyses are hypothesis-generating and were not adjusted for multiplicity.

Ventricular runs/non-sustained ventricular tachycardia were similarly infrequent (5.1%, 5.6%, 6.1%, 6.3%, and 3.1%; Cochran Q *p* = 1.000, all paired *p* = 1.000) (Table 2).

Hypertension prevalence increased from 45% at baseline to 71% at 12 months and 70% at the nominal 36-month visit. Among 22 patients without baseline hypertension, 9 (41%) developed incident hypertension. The mean number of antihypertensive drug classes increased from 1.08 at baseline to 1.75 by 6 months and remained elevated thereafter.

## Discussion

In this prospective observational cohort, serial imaging during chronic ibrutinib therapy showed a coherent pattern of LA remodeling: modest enlargement, worsening reservoir and contractile function, declining atrial-wall tissue velocity, and lower absolute LV GLS, while conduit strain and conventional filling indices were largely stable. Incident AF occurred in five patients. AF-subgroup differences involved only four events by 12 months (should be considered hypothesis-generating). Although statistically robust, the absolute LAVI increments were modest (+1.5 to +2.6 mL/m²; SRM 0.48–0.85), and this study was not designed to establish a minimal clinically important change. The principal signal is therefore the concordant deterioration across multiple deformation and atrial-function measures rather than the magnitude of any single volumetric change.

The cumulative AF incidence of 12.5% in our cohort is concordant with a real-world AF burden associated with chronic ibrutinib therapy. In registration-trials and registries, AF incidence was generally reported in the range of approximately 4%–16%, with cumulative incidence increasing with longer exposure (2–6, 9, 23–26). In prospective cardio-oncology cohorts with systematic surveillance, substantially higher rates have been observed, likely reflecting more active detection of asymptomatic events (23, 26). The most important contribution of the present study is that it moves the focus beyond the binary occurrence of AF toward the underlying atrial phenotype. We observed a pattern of progressive LA remodeling, with increasing LAVI and worsening LA reservoir strain, LA contractile strain, and LA lateral wall indices over time, accompanied by deterioration in left ventricular GLS despite preserved conventional ejection fraction. This could indicate that subclinical atrial and ventricular dysfunction may be detectable earlier and more sensitively by deformation imaging than by standard echocardiographic measures alone (17, 27).

Previous studies established that pretreatment LA enlargement or impaired LA mechanics may identify patients at higher risk of AF during ibrutinib therapy (18, 26, 28, 29). The present study's incremental contribution is prospective, repeated characterization of component-wise LA mechanics and volume over prolonged follow-up. It should therefore be interpreted as a longitudinal phenotyping study. The subgroup findings of our study reinforce this interpretation. Patients who developed AF by 12 months had larger baseline LAVI and persistently larger LA volumes during follow-up, together with lower LA conduit strain at 6 months. Taken together, these data support the concept that AF associated with ibrutinib therapy is facilitated by an atrial substrate that is already abnormal or becomes abnormal early during therapy. This interpretation is aligned with earlier prospective studies identifying hypertension, older age, and larger LA size as predictors of AF associated with chronic ibrutinib therapy (18, 26–30). In contrast, we did not observe a clear monotonic increase in supraventricular or ventricular premature beats burden, suggesting that the dominant signal in our cohort was progressive substrate abnormality rather than increasing ectopic activity (12, 31).

A plausible mechanistic framework for these findings is provided by preclinical data showing that ibrutinib-related AF is largely mediated by off-target cardiac effects rather than by BTK inhibition itself (12–14). Xiao et al. demonstrated that ibrutinib exposure in mice induced AF susceptibility, LA enlargement, fibrosis, and inflammation, and identified inhibition of C-terminal Src kinase as a key mechanistic mediator (28). These data provide biological support for the progressive LA enlargement and worsening mechanical function observed in our cohort suggesting that echocardiographic remodeling may represent an intermediate phenotype. This cautious interpretation is consistent with recent pharmacovigilance data suggesting that, after adjustment for confounders, AF associated with ibrutinib is not clearly dose dependent (32).

Another relevant observation in our study was the progressive increase in hypertension prevalence and antihypertensive therapy. Nearly 41% of patients without baseline hypertension developed incident hypertension, and the mean number of antihypertensive drug classes increased early during follow-up and remained elevated. Because hypertension increased during follow-up and could contribute to remodeling, we repeated the mixed models with contemporaneous hypertension as a time-varying covariate. The principal visit estimates were materially unchanged. This does not exclude mediation or residual confounding, particularly because hypertension may lie on the causal pathway and the cohort was small. This is important because hypertension is both a recognized toxicity of ibrutinib and a likely contributor to atrial remodeling (5, 10, 26, 33–35). In a large cohort study, new or worsened hypertension occurred in most patients after ibrutinib initiation and was associated with subsequent major cardiovascular events (34, 35). The higher antihypertensive treatment burden among patients who developed AF in our cohort further supports the possibility that blood-pressure elevation and atrial arrhythmogenesis are closely linked in this setting. Clinically, these findings argue for proactive blood-pressure monitoring and management as an integral part of cardio-oncology follow-up (27, 33–35). These findings are specific to ibrutinib and should not be extrapolated directly to newer BTK inhibitors. Head-to-head trials of acalabrutinib and zanubrutinib versus ibrutinib, and recent randomized data for pirtobrutinib, report lower rates of AF and/or hypertension with the more selective agents (36–38). Whether similar serial LA-mechanics changes occur with these agents remains unknown.

Our study findings could have direct clinical implications. Baseline cardiovascular assessment before the start of ibrutinib therapy should probably extend beyond symptom review, office blood pressure measurement, and left ventricular ejection fraction. In patients planned for long-term therapy, assessment of LA size and, ideally LA strain may help identify a higher-risk atrial phenotype. Also, during follow-up, structured and more strict surveillance for hypertension and rhythm disturbances appears warranted, especially during the first year, but also beyond it in order to maintain BTKi therapy whenever possible (18, 27, 30).

### Limitations

This study has several limitations. It was a small, single-arm observational cohort without an untreated or alternative-BTK-inhibitor comparator, so the changes observed during therapy cannot be attributed specifically to ibrutinib, because aging, hematologic disease, previous treatment, and incident hypertension may have contributed. The cardiovascular exclusion criteria produced a selected cohort and limit generalizability to older, multimorbid (especially cardiovascular) patients in routine practice. Only five AF events occurred, making all AF-subgroup analyses exploratory. AF surveillance used symptom-triggered ECGs and scheduled 24–72-hour Holter-ECG monitoring, so asymptomatic paroxysmal episodes may have been missed and event timing was interval-censored. Missingness increased over follow-up, although mixed-model and complete-case analyses were concordant. Exact treatment-line details, cumulative exposure, and longitudinal hematologic response were not recorded. Finally, multiple endpoints were assessed, false-discovery-rate correction was applied to the clearly defined central remodeling family, while secondary analyses remain exploratory.

## Conclusion

In this selected, longitudinal, observational cohort wih long-term follow-up, chronic ibrutinib therapy was accompanied by modest LA enlargement and consistent deterioration in several LA deformation indices, together with increasing hypertension burden. Serial LA mechanics may be useful for characterizing subclinical atrial remodeling, but the present data do not establish an ibrutinib-specific causal effect or a validated AF-prediction strategy. Controlled studies, including cohorts receiving newer BTK inhibitors, are required before these measurements can guide treatment decisions.

## Data Availability

The raw data supporting the conclusions of this article will be made available by the authors, without undue reservation.
